# First-Pass Success of Video Laryngoscope Compared With Direct Laryngoscope in Intubations Performed by Residents in the Emergency Department

**DOI:** 10.7759/cureus.47563

**Published:** 2023-10-24

**Authors:** Akihiko Sugaya, Keiko Naito, Tadahiro Goto, Yusuke Hagiwara, Hiroshi Okamoto, Hiroko Watase, Kohei Hasegawa

**Affiliations:** 1 Emergency and Critical Care Medicine, Tokyo Bay Urayasu Ichikawa Medical Center, Chiba, JPN; 2 Clinical Epidemiology and Health Economics, School of Public Health, University of Tokyo, Tokyo, JPN; 3 Paediatric Emergency and Critical Care Medicine, Tokyo Metropolitan Children's Medical Center, Tokyo, JPN; 4 Critical Care Medicine, St. Luke's International Hospital, Tokyo, JPN; 5 Emergency Medicine and General Internal Medicine, School of Medicine, Fujita Health University, Aichi, JPN; 6 Emergency Medicine, Massachusetts General Hospital, Boston, USA

**Keywords:** video laryngoscope, emergency medicine resident, intubation complication, airway intubation, airway management

## Abstract

Background: The video laryngoscope (VL) has been widely used for intubation in the emergency department (ED). However, their effectiveness remains controversial, particularly among airway management performed by residents in the ED.

Methods: We aimed to examine whether the use of VL, compared to a direct laryngoscope (DL), was associated with higher first-attempt intubation success among intubations performed by residents in the ED. This is a secondary analysis of the data from a prospective, observational, multicentre study of 15 Japanese EDs from April 2012 through March 2020. We included all adult patients who underwent intubation with VL or DL by residents (postgraduate years ≤5) in the ED. The outcome measures were first-pass success and intubation-related adverse events (overall, major, and minor adverse events). To determine the association of VL use with each of the outcomes, we constructed logistic regression models with generalized estimating equations to account for patients clustering within the ED, adjusting for patient demographics, primary indications, intubation difficulty, and intubation methods.

Results: Of 5,261 eligible patients who underwent an initial intubation attempt by residents, 1,858 (35%) patients were attempted with VL. Intubations performed with VL had a non-significantly higher first-pass success rate than those with DL (77% vs. 64%; unadjusted odds ratio (OR)=1.20; 95% CI=0.87-1.65; P=0.27). This association was significant after adjustment for potential confounders (adjusted OR, 1.33; 95% CI, 1.06-1.67; P=0.01). As for adverse events, the use of VL was associated with a lower rate of any (adjusted OR=0.67; 95% CI=0.51-0.86; P=0.002) and minor (adjusted OR=0.69; 95% CI=0.55-0.87; P=0.002) adverse events.

Conclusion: The use of VL was associated with a higher first-attempt success rate and a lower rate of any adverse events compared to that with DL among intubations performed by residents in the EDs.

## Introduction

Tracheal intubation is a critical intervention performed in the emergency department (ED). The first-attempt intubation success (first-pass success) rate in the ED remains suboptimal, ranging from 63% to 83%, as reported in studies using large registries [1-3]. In addition, with the limited resources and an urgent situation, difficult intubations more frequently occurred in the ED than in the operating room setting (13% vs. 5.8%) [4,5]. Regardless, physicians should achieve first-pass success in such settings as multiple intubation attempts lead to adverse events [6,7] and poor in-hospital outcomes [8].

In recent years, the video laryngoscope (VL) is more commonly used for intubation in critically ill patients in the ED [9]. VL provides better glottic visualization and less oesophageal intubation than the direct laryngoscope (DL) [10-12]. The utility of VL compared to DL in improving first-pass success rates and reducing adverse events was previously unclear; however, in recent times, there has been growing support for the effectiveness of VL [12-15]. Nonetheless, the utility of VL in endotracheal intubation performed by resident physicians remains uncertain. In clinical education for residents, tracheal intubation is one of the most important procedures that residents should experience during their training program. Despite its importance, the effectiveness of VL on intubation performance among intubations performed by residents in the ED remains to be elucidated.

To address the knowledge gap in the literature, we used data from a prospective multicentre study to investigate whether the use of VL compared to the use of DL was associated with a higher first-pass success rate and lower adverse event rate of intubation by residents in EDs. A better understanding of this crucial issue in emergency airway management will inform physicians to develop optimal curricula for residency training.

## Materials and methods

Study design and setting

This is a secondary analysis of the data from a prospective, observational, multicentre study of emergency airway management, the second Japanese Emergency Airway Network (JEAN-2) study. The study design, setting, methods of measurement, and measured variables have been reported previously [16-19]. In short, the JEAN-2 study was a consortium of 15 academic and community medical centers from different geographic regions across Japan. The participating institutions included 12 level-I and three level-II equivalent trauma centers with a median ED census of 26,692 patient visits per year (range=1,078-60,101 per year). All 15 EDs were staffed by emergency attending physicians and affiliated with emergency medicine residency programs. Intubations were performed by transitional-year residents (postgraduate years one and two), emergency medicine residents (both of which were at the discretion of attending physicians), attending emergency physicians, or other specialties (e.g., surgery, anesthesia, pediatrics). The institutional review board of each participating hospital approved the study protocol with a waiver of informed consent prior to data collection.

Study participants

We included all adult patients (aged ≥18 years) who underwent tracheal intubation in the ED by postgraduate residents (defined as a doctor who has started working within five years after graduating from medical school, which included transitional-year residents, emergency medicine residents, and other specialty residents) from April 2012 through March 2020. We excluded patients intubated by other than VL and DL or by adjunctive devices, or those with missing data on the patient’s age, body mass index (BMI), modified look-evaluate-Mallampati- obstruction-neck (LEMON), intubation devices, intubator’s postgraduate year, or specialty.

Data collection and processing

The intubators filled out a standardized data collection form on each intubation. Collected information included the patient’s demographics (age, sex, height, and weight), primary indication for intubation, components of modified LEMON criteria [20,21], all medications used for intubation, intubation methods (no medication, rapid sequence intubation (RSI), sedation without paralysis, and others), ED visit year, devices of intubation, intubator’s level of training and specialty, intubation success or failure, the number of intubation attempts, vital signs (at pre-intubation, one minute and 30 minutes after intubation), and adverse events. The modified LEMON criteria were composed of external looks, the distance between the incisor teeth, the distance between the hyoid bone and the chin, airway obstruction, and neck immobility [20,21]. The Japanese Emergency Medicine Network (JEMNet) coordinating center and site investigator at each ED reviewed the data forms. If information was missing or contained inconsistencies, the data form was returned to the intubator for completion or the site investigator asked the intubator for the airway management details.

Primary exposure

The primary exposure was intubation devices - VL and DL - without adjunctive devices (e.g., bougie). VL was divided into C-MAC, McGrath, or other VLs (e.g., Airway Scope (AWS®, Pentax Corp., Tokyo, Japan) and GlideScope (Saturn Biomedical System Inc., Burnaby, BC, Canada)).

Outcome measures

The primary outcome was first-pass success defined as successful intubation on the first attempt. We have not collected data on time for intubation. The secondary outcomes were intubation-related adverse events, including major and minor adverse events. Major adverse events were defined as cardiac arrest, dysrhythmia, hypotension (systolic blood pressure <90 mmHg), hypoxia (pulse oximetry saturation <90%), and oesophageal intubation with delayed recognition [6,9,17,18,22]. Minor adverse events were defined as regurgitation, airway trauma, dental or lip trauma, mainstem bronchial intubation, and oesophageal intubation recognized immediately [6,9,18,22].

Statistical analysis

First, we compared the patients and airway management characteristics between VL and DL groups, using the Mann-Whitney U test and chi-square test as appropriate. Second, to investigate the association of devices (VL and DL) with each of the outcomes, we constructed the unadjusted and adjusted logistic regression models with generalized estimating equations (GEE) to account for patients clustering within the ED. In the multivariable models, we adjusted for age (<65, and ≥65 years old), sex, BMI (<25.0, 25.0-29.9, and ≥30.0 kg/m^2^), primary indication (medical indication, traumatic indication, and cardiac arrest), modified LEMON, premedication use, intubation methods (no medication, RSI, sedation without paralysis, and others), and ED visit year. We selected these potential confounders based on clinical plausibility and a priori knowledge [6,14,17]. We also fit the unadjusted and adjusted multivariable logistic regression models with GEE in which VL was divided into C-MAC, McGrath, and other VLs (Airway Scope and GlideScope).

We conducted a series of sensitivity analyses to examine the robustness of our inference. First, we repeated the analyses for the outcomes with stratification by 1) BMI category (<25.0, 25.0-29.9, and ≥30.0 kg/m^2^), 2) primary indication (cardiac arrest and non-cardiac arrest), 3) modified LEMON (0 and ≥1), 4) intubation methods (RSI and non-RSI), and 5) intubator’s specialty (transitional-year residents, emergency medicine residents, and other specialty residents). Lastly, we repeated the analysis by including intubations attempted by VL or DL with the use of a bougie. All P values of <0.05 were regarded as statistically significant. All statistical analyses were performed with STATA 14.1 (StataCorp, College Station, TX) and the EZR software (Saitama Medical Centre, Jichi Medical University, Saitama, Japan) - a graphical user interface for R (version 4.0.1; R Foundation for Statistical Computing, Vienna, Austria) [23].

## Results

**Figure 1:**
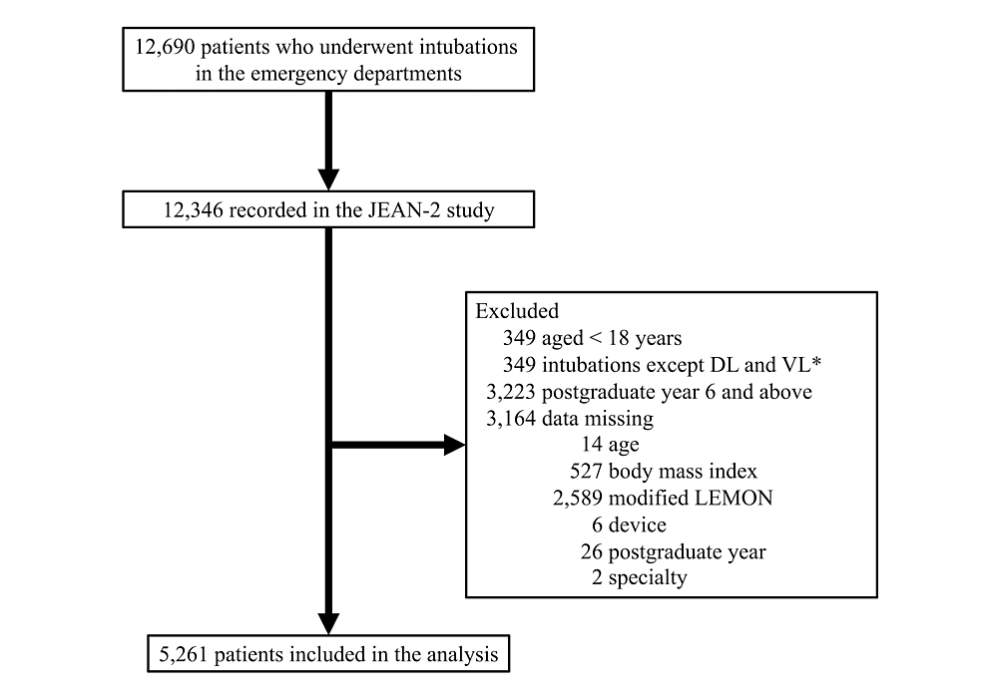
Study flow During the study period, the JEAN-2 study recorded a total of 12,346 patients who underwent emergency airway management at one of the 15 emergency departments (capture rate, 98.5%). Of these, 5,261 patients were eligible for the current analysis. Abbreviation: JEAN, Japanese Emergency Airway Network * We excluded fiberoptic intubation, lighted-style-assisted tracheal intubation, nasal intubation, cricothyrotomy, the use of supraglottic devices, and the use of bougie.

During the study period, there were 12,690 patients who underwent intubation in the ED. The JEAN-2 study recorded 12,346 patients (capture rate, 98.5%). After removing non-eligible patients (Figure 1), the current study included 5,261 patients who underwent an intubation attempt by resident physicians in the ED. Table 1 summarizes the baseline characteristics and airway management of patients. Overall, the median age was 72 years (IQR=59-81 years), and 38% were female. The VL and DL groups had some differences in the patient and airway management characteristics. For example, the VL group was younger and more likely to have a medical indication and to be intubated with RSI (P<0.05).

**Table 1:** Baseline characteristics and airway management of 5,261 patients who underwent tracheal intubation by residents in the emergency department, according to intubation devices. Abbreviations: DL, direct laryngoscope; IQR, interquartile range; VL, video laryngoscope Data are expressed as numbers (percentages) unless otherwise indicated. * Type of VL: C-MAC (n=1,360), McGrath (n=380), and other VLs (n=118). † Premedication includes fentanyl and lidocaine. ‡ Others include intubations using paralytics and analgesics only.

Variables	Overall (n=5,261)	VL* (n=1,858)	DL (n=3,403)	P value
Patient characteristics				
Age (year), median (IQR)	72 (59-81)	70 (56-79)	74 (61-82)	<0.001
Age ≥65 years	3,511 (67)	1,137 (61)	2,374 (70)	<0.001
Female sex	2,000 (38)	717 (39)	1,283 (38)	0.55
Body mass index (kg/m^2^)				0.04
<25.0	4,113 (78)	1,420 (76)	2,693 (79)	
25.0-29.9	915 (17)	342 (18)	573 (17)	
≥30.0	233 (4)	96 (5)	137 (4)	
Primary indication				<0.001
Medical indication	2,885 (55)	1,263 (68)	1,622 (48)	
Traumatic indication	415 (8)	236 (13)	179 (5)	
Cardiac arrest	1,961 (37)	359 (19)	1,602 (47)	
Airway management				
Anticipated difficult intubation				
≥1 modified LEMON	2,546 (48)	899 (48)	1,647 (48)	0.99
Premedication use†	1,190 (23)	911 (49)	279 (8)	<0.001
Intubation methods				<0.001
No medication	2,632 (50)	506 (27)	2,126 (62)	
Rapid sequence intubation	1,897 (36)	1,082 (58)	815 (24)	
Sedation without paralysis	541 (10)	196 (11)	345 (10)	
Others‡	191 (4)	74 (4)	117 (3)	
ED visit year				<0.001
2012	474 (9)	58 (3)	416 (12)	
2013	877 (17)	136 (7)	741 (22)	
2014	799 (15)	179 (10)	620 (18)	
2015	757 (14)	226 (12)	531 (16)	
2016	792 (15)	334 (18)	458 (13)	
2017	681 (13)	335 (18)	346 (10)	
2018	430 (8)	262 (14)	168 (5)	
2019	376 (7)	268 (14)	108 (3)	
2020	75 (1)	60 (3)	15 (0.4)	

**Figure 2:**
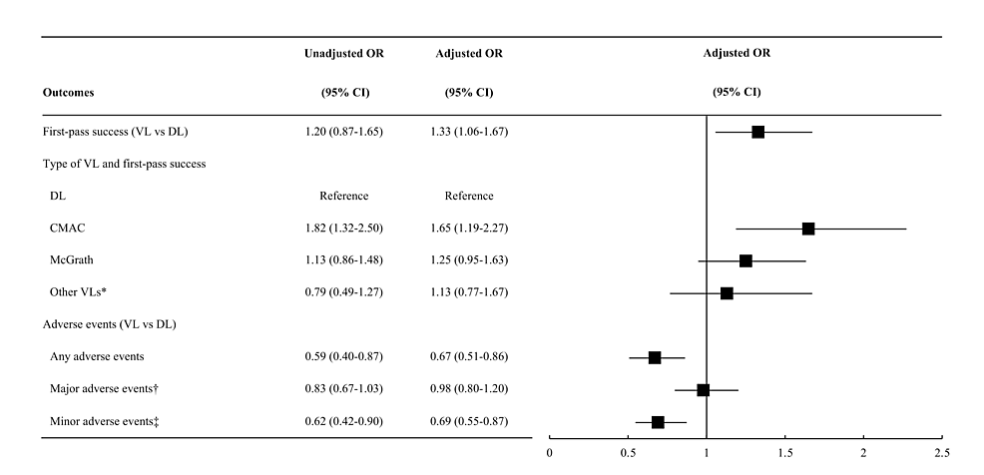
Unadjusted and adjusted associations of video laryngoscope with first-pass success and adverse events in the emergency department Abbreviations: CI, confidence interval; DL, direct laryngoscope; OR, odds ratio; VL, video laryngoscope Multivariable logistic regression model with generalized estimating equations (GEE) adjusting for age, sex, body mass index, primary indications, modified LEMON, premedication use, intubation methods, and ED visit year, as well as accounting for within-ED clustering. * Other video laryngoscopes include Airway Scope and GlideScope. † Major adverse events include cardiac arrest, dysrhythmia, hypotension, hypoxia, and oesophageal intubation with delayed recognition. ‡ Minor adverse events include regurgitation, airway trauma, dental or lip trauma, mainstem bronchial intubation, and oesophageal intubation recognized immediately.

Overall, the first-pass success rate was 69%. The VL group had a non-significantly higher first-pass success rate compared to the DV group (77% vs. 64%; unadjusted OR=1.20; 95% CI=0.87-1.65; P=0.27). After adjusting for the confounders, this association became significant (adjusted OR=1.33; 95% CI=1.06-1.67; P=0.01; Table 2 and Figure 2). Among the VLs, a significantly higher first-pass success rate was observed in C-MAC (adjusted OR=1.65; 95% CI=1.19-2.27; P=0.002).

**Table 2:** Unadjusted and adjusted odds ratio for first-pass success* Abbreviations: CI, confidence interval; DL, direct laryngoscope; VL, video laryngoscope * The unadjusted and adjusted logistic regression models with generalized estimating equations (GEE) to account for patients clustering within the ED. † Type of VL: C-MAC (n=1,360), McGrath (n=380), and other VLs (n=118). ‡ Premedication includes fentanyl and lidocaine. § Others include intubations using paralytics and analgesics only.

Model and variable	Odds ratio (95% CI)	P value
Unadjusted model		
VL (vs DL)†	1.20 (0.87-1.65)	0.27
Adjusted model		
VL (vs DL)†	1.33 (1.06-1.67)	0.01
Covariates		
Age ≥65 years	1.12 (1.00-1.26)	0.06
Female sex	1.14 (1.00-1.31)	0.06
Body mass index (kg/m^2^)		
<25.0	Reference	
25.0-29.9	0.84 (0.69-1.01)	0.06
≥30.0	0.60 (0.46-0.79)	<0.001
Primary indication		
Medical indication	Reference	
Traumatic indication	0.76 (0.65-0.89)	0.001
Cardiac arrest	1.24 (1.04-1.47)	0.02
Airway management		
Anticipated difficult intubation		
≥1 modified LEMON	0.59 (0.52-0.67)	<0.001
Premedication use‡	1.02 (0.87-1.20)	0.81
Intubation methods		
No medication	Reference	
Rapid sequence intubation	1.87 (1.42-2.46)	<0.001
Sedation without paralysis	0.73 (0.56-0.96)	0.02
Others§	1.21 (0.81-1.80)	0.36
ED visit year		
2012	Reference	
2013	0.96 (0.82-1.13)	0.64
2014	1.14 (0.96-1.34)	0.14
2015	1.04 (0.72-1.51)	0.82
2016	1.03 (0.82-1.30)	0.78
2017	1.00 (0.80-1.25)	0.99
2018	1.33 (1.00-1.78)	0.05
2019	1.22 (0.90-1.64)	0.20
2020	1.15 (0.80-1.64)	0.46

Overall, the rate of intubation-related adverse events was 10%. The use of VL was also associated with a significantly lower rate of any adverse events (adjusted OR=0.67; 95% CI=0.51-0.86; P=0.002). This association was mainly driven by the significant association with the lower minor adverse events (adjusted OR=0.69; 95% CI=0.55-0.87; P=0.002; Figure 2).

Sensitivity analysis

The associations between VL use and higher first-pass success were also observed in the sensitivity analyses (Table 3). For example, the use of VL was associated with a significantly higher first-pass success rate in the groups of BMI <25.0 kg/m^2^ (adjusted OR=1.35; 95% CI=1.05-1.73; P=0.02), non-cardiac arrest (adjusted OR=1.33; 95% CI=1.04-1.70; P=0.03), and modified LEMON=0 (adjusted OR=1.60; 95% CI=1.22-2.09; P=0.001). Additionally, a similar association was found in the group of RSI use (adjusted OR=1.91; 95% CI=1.58-2.31; P<0.001). Likewise, the use of VL (including the use of bougie) was also associated with a significantly higher first-pass success rate (adjusted OR=1.40; 95% CI=1.16-1.69; P=0.001; Table 4).

**Table 3:** Adjusted odds ratio for first-pass success, according to subgroups Abbreviations: CI, confidence interval; DL, direct laryngoscope; OR, odds ratio; PGY, postgraduate year; RSI, rapid sequence intubation; VL, video laryngoscope * Multivariable logistic regression model with generalized estimating equations (GEE) adjusting for age, sex, primary indications, modified LEMON, premedication use, intubation methods, and ED visit year as well as accounting for within-ED clustering. † Multivariable logistic regression model with GEE adjusting for age, sex, body mass index, modified LEMON, premedication use, intubation methods, and ED visit year as well as accounting for within-ED clustering. ‡ Multivariable logistic regression model with GEE adjusting for age, sex, body mass index, primary indications, premedication use, intubation methods, and ED visit year as well as accounting for within-ED clustering. § Multivariable logistic regression model with GEE adjusting for age, sex, body mass index, primary indications, modified LEMON, premedication use, and ED visit year as well as accounting for within-ED clustering. || Multivariable logistic regression model with GEE adjusting for age, sex, body mass index, primary indications, modified LEMON, premedication use, intubation methods, and ED visit year as well as accounting for within-ED clustering.

Subgroup	Adjusted OR (VL vs. DL) in each subgroup (95% CI)	P value
Body mass index (kg/m^2^)*		
<25.0	1.35 (1.05-1.73)	0.02
25.0-29.9	1.59 (0.86-2.94)	0.14
≥30	1.08 (0.62-1.90)	0.79
Primary indication†		
Cardiac arrest	1.37 (0.98-1.90)	0.06
Non-cardiac arrest	1.33 (1.04-1.70)	0.03
Modified LEMON‡		
0	1.60 (1.22-2.09)	0.001
≥1	1.28 (0.97-1.70)	0.08
Intubation method§		
RSI	1.91 (1.58-2.31)	<0.001
Non-RSI	1.21 (0.93-1.60)	0.16
Intubator’s specialty||		
Transitional-year residents (PGY 1-2)	1.18 (0.95-1.47)	0.14
Emergency medicine residents (PGY 3-5)	1.20 (0.71-2.06)	0.49
Other specialty residents (PGY 3-5 and non-emergency medicine residents)	0.55 (0.38-0.80)	0.002

**Table 4:** Unadjusted and adjusted associations of video laryngoscope that include bougie use with first-pass success and adverse events in the emergency department Abbreviations: CI, confidence interval; DL, direct laryngoscope; OR, odds ratio; VL, video laryngoscope; C-MAC, cipher-based message authentication codes Multivariable logistic regression model with generalized estimating equations (GEE) adjusting for age, sex, body mass index, primary indications, modified LEMON, premedication use, intubation methods, and ED visit year as well as accounting for within-ED clustering. * Other video laryngoscopes include Airway Scope and GlideScope. † Major adverse events include cardiac arrest, dysrhythmia, hypotension, hypoxia, and oesophageal intubation with delayed recognition. ‡ Minor adverse events include regurgitation, airway trauma, dental or lip trauma, mainstem bronchial intubation, and oesophageal intubation recognized immediately.

Outcomes	Unadjusted OR (95% CI)	P value	Adjusted OR (95% CI)	P value
First-pass success (VL vs DL)	1.26 (0.92-1.72)	0.15	1.40 (1.16-1.69)	0.001
Type of VL and first-pass success				
DL	Reference		Reference	
C-MAC	1.81 (1.31-2.50)	<0.001	1.65 (1.19-2.27)	0.002
McGrath	1.24 (0.99-1.54)	0.06	1.38 (1.13-1.68)	0.001
Other VLs*	0.77 (0.46-1.29)	0.32	1.09 (0.69-1.72)	0.70
Adverse events (VL vs DL)				
Any adverse events	0.54 (0.40-0.74)	<0.001	0.62 (0.50-0.78)	<0.001
Major adverse events†	0.81 (0.63-1.04)	0.09	0.98 (0.77-1.25)	0.87
Minor adverse events‡	0.57 (0.42-0.78)	<0.001	0.65 (0.52-0.82)	<0.001

The associations between VL use and lower rates of intubation-related adverse events were also observed in the sensitivity analyses (Tables 5-7). For example, the use of VL was associated with a significantly lower rate of any adverse events in the groups of BMI <25.0 kg/m^2^ (adjusted OR=0.64; 95% CI=0.50-0.83; P=0.001) and transitional-year residents (adjusted OR=0.44; 95% CI=0.31-0.61; P<0.001). Similarly, this association remained significant after stratification by primary indication, modified LEMON, and RSI use (all P<0.05). Lastly, the use of VL (including the use of bougie) was also associated with a significantly lower adverse event rate (adjusted OR=0.62; 95% CI=0.50-0.78; P<0.001; Table 4).

**Table 5:** Adjusted associations between video laryngoscope and any intubation-related adverse events, according to subgroups Abbreviations: CI, confidence interval; DL, direct laryngoscope; OR, odds ratio; PGY, postgraduate year; RSI, rapid sequence intubation; VL, video laryngoscope * Multivariable logistic regression model with generalized estimating equations (GEE) adjusting for age, sex, primary indications, modified LEMON, premedication use, intubation methods, and ED visit year as well as accounting for within-ED clustering. † Multivariable logistic regression model with GEE adjusting for age, sex, body mass index, modified LEMON, premedication use, intubation methods, and ED visit year as well as accounting for within-ED clustering. ‡ Multivariable logistic regression model with GEE adjusting for age, sex, body mass index, primary indications, premedication use, intubation methods, and ED visit year as well as accounting for within-ED clustering. § Multivariable logistic regression model with GEE adjusting for age, sex, body mass index, primary indications, modified LEMON, premedication use, and ED visit year as well as accounting for within-ED clustering. || Multivariable logistic regression model with GEE adjusting for age, sex, body mass index, primary indications, modified LEMON, premedication use, intubation methods, and ED visit year as well as accounting for within-ED clustering.

	Adjusted OR (VL vs. DL) (95% CI)	P value
Body mass index (kg/m^2^)*		
<25.0	0.64 (0.50-0.83)	0.001
25.0-29.9	0.79 (0.57-1.11)	0.18
≥30	1.01 (0.63-1.60)	0.97
Primary indication†		
Cardiac arrest	0.54 (0.30-0.98)	0.04
Non-cardiac arrest	0.66 (0.54-0.81)	<0.001
Modified LEMON‡		
0	0.50 (0.37-0.67)	<0.001
≥1	0.70 (0.54-0.90)	0.005
Intubation method§		
RSI	0.57 (0.44-0.74)	<0.001
Non-RSI	0.68 (0.52-0.89)	0.005
Intubator’s specialty||		
Transitional-year residents (PGY 1-2)	0.44 (0.31-0.61)	<0.001
Emergency medicine residents (PGY 3-5)	0.89 (0.52-1.52)	0.68
Other specialty residents (PGY 3-5 and non-emergency medicine residents)	0.70 (0.31-1.60)	0.40

**Table 6:** Adjusted associations between video laryngoscope and major intubation-related adverse events, according to subgroups Abbreviations: CI, confidence interval; DL, direct laryngoscope; OR, odds ratio; PGY, postgraduate year; RSI, rapid sequence intubation; VL, video laryngoscope * Multivariable logistic regression model with generalized estimating equations (GEE) adjusting for age, sex, primary indications, modified LEMON, premedication use, intubation methods, and ED visit year as well as accounting for within-ED clustering. † Multivariable logistic regression model with GEE adjusting for age, sex, body mass index, modified LEMON, premedication use, intubation methods, and ED visit year as well as accounting for within-ED clustering. ‡ Multivariable logistic regression model with GEE adjusting for age, sex, body mass index, primary indications, premedication use, intubation methods, and ED visit year as well as accounting for within-ED clustering. § Multivariable logistic regression model with GEE adjusting for age, sex, body mass index, primary indications, modified LEMON, premedication use, and ED visit year as well as accounting for within-ED clustering. || Multivariable logistic regression model with GEE adjusting for age, sex, body mass index, primary indications, modified LEMON, premedication use, intubation methods, and ED visit year as well as accounting for within-ED clustering.

	Adjusted OR (VL vs. DL) (95% CI)	P value
Body mass index (kg/m^2^)*		
<25.0	0.94 (0.77-1.16)	0.59
25.0-29.9	1.29 (0.90-1.85)	0.17
≥30	7.22 (1.70-30.75)	0.007
Primary indication†		
Cardiac arrest	0.69 (0.35-1.36)	0.29
Non-cardiac arrest	0.97 (0.73-1.29)	0.84
Modified LEMON‡		
0	0.77 (0.63-0.94)	0.01
≥1	1.06 (0.81-1.39)	0.66
Intubation method§		
RSI	1.01 (0.64-1.59)	0.96
Non-RSI	0.91 (0.66-1.26)	0.59
Intubator’s specialty||		
Transitional-year residents (PGY 1-2)	0.66 (0.40-1.09)	0.10
Emergency medicine residents (PGY 3-5)	1.13 (0.66-1.96)	0.65
Other specialty residents (PGY 3-5 and non-emergency medicine residents)	0.74 (0.34-1.61)	0.45

**Table 7:** Adjusted associations between video laryngoscope and minor intubation-related adverse events, according to subgroups Abbreviations: CI, confidence interval; DL, direct laryngoscope; OR, odds ratio; PGY, postgraduate year; RSI, rapid sequence intubation; VL, video laryngoscope * Multivariable logistic regression model with generalized estimating equations (GEE) adjusting for age, sex, primary indications, modified LEMON, premedication use, intubation methods, and ED visit year as well as accounting for within-ED clustering. † Multivariable logistic regression model with GEE adjusting for age, sex, body mass index, modified LEMON, premedication use, intubation methods, and ED visit year as well as accounting for within-ED clustering. ‡ Multivariable logistic regression model with GEE adjusting for age, sex, body mass index, primary indications, premedication use, intubation methods, and ED visit year as well as accounting for within-ED clustering. § Multivariable logistic regression model with GEE adjusting for age, sex, body mass index, primary indications, modified LEMON, premedication use, and ED visit year as well as accounting for within-ED clustering. || Multivariable logistic regression model with GEE adjusting for age, sex, body mass index, primary indications, modified LEMON, premedication use, intubation methods, and ED visit year as well as accounting for within-ED clustering.

	Adjusted OR (VL vs. DL) (95% CI)	P value
Body mass index (kg/m^2^)*		
<25.0	0.66 (0.52-0.84)	0.001
25.0-29.9	0.80 (0.57-1.12)	0.20
≥30	1.06 (0.72-1.56)	0.76
Primary indication†		
Cardiac arrest	0.57 (0.31-1.05)	0.07
Non-cardiac arrest	0.69 (0.58-0.81)	<0.001
Modified LEMON‡		
0	0.53 (0.39-0.71)	<0.001
≥1	0.72 (0.58-0.90)	0.003
Intubation method§		
RSI	0.61 (0.48-0.77)	<0.001
Non-RSI	0.71 (0.55-0.91)	0.007
Intubator’s specialty||		
Transitional-year residents (PGY 1-2)	0.47 (0.35-0.63)	<0.001
Emergency medicine residents (PGY 3-5)	0.89 (0.52-1.52)	0.68
Other specialty residents (PGY 3-5 and non-emergency medicine residents)	0.72 (0.32-1.61)	0.42

## Discussion

Based on the prospective multicentre data of 5,261 ED patients intubated by resident physicians, intubation with VL achieved a significantly higher first-pass success rate than that with DL. In particular, intubation with C-MAC had the highest rate. In addition, we also found that the use of VL was associated with significantly fewer overall adverse events. The inference was consistent across the different statistical assumptions and most patient subgroups.

Few studies have reported the effectiveness of VL in intubations by residents. Within the sparse literature, a systematic review has reported that VL used outside the operating room setting by novices and trainees was associated with a higher first-pass success rate compared to DL [12]. In an ED setting, a single-center study has shown that VL use was associated with a higher first-pass success rate compared to DL [14]. Our findings are consistent with these previous studies. In contrast, research has reported that the association of VL and first-pass success rate differs by type of VL [13]. For example, a secondary analysis of a prospective multicentre cohort study (the National Emergency Airway Registry-Ⅲ) has reported that only C-MAC was associated with a higher first-attempt success rate, while overall VL did not have a significantly higher or lower rate of intubation success [13]. The apparent differences between the studies may be attributable to the differences in the study design, target population, data collection methods, analysis, or any combination of these factors. The current large prospective study builds on these earlier reports and extends them by demonstrating a robust relationship between VL use with a higher first-pass success rate and a lower adverse event rate.

There are several possible explanations for the observed relationship of VL use with first-pass success. First, the use of VL contributes to better glottic visualization [11]. Critically ill patients have various difficult intubation factors (e.g., difficult airways and inadequate preoxygenation) [24]. Improved glottic visualization by VL may have enabled residents to intubate such patients easily. In addition, the advantage of C-MAC is the use of a standard Macintosh-type blade, which may be familiar to physicians [25]. Second, the dynamic supervision by attending physicians through the shared VL monitor may have also contributed to superior intubation outcomes [26]. Our data also demonstrated that the use of VL was not associated with a lower rate of major intubation-related adverse events but associated with any and minor adverse events, consistent with previous reports showing the association between the use of VL and reduced oesophageal intubations [10,12]. These findings can be explained, at least partially, by the improved intubation maneuvers with the use of DL because novice physicians use a conventional DL by applying extra force on the oral structures [27,28], thereby leading to airway trauma. Furthermore, the better glottic visualization with VL may have reduced the oesophageal intubation. Moreover, a higher first-pass success rate with VL likely led to fewer adverse events as multiple intubation attempts constitute a major risk factor for adverse events [6]. Lastly, these potential mechanisms are not mutually exclusive. Notwithstanding the complexity of the mechanisms, the observed superiority of VL in ED intubations supports the use of VL as the first device of choice for resident physicians in the ED.

Limitations

This study has several potential limitations. First, the passive surveillance system adopted in this study may have led to self-reporting bias, resulting in a possible overestimation of the first-attempt success rate and underestimation of the adverse event rate. Regardless, we believe that a capture rate of 98.5% with standardized data collection ensured the quality of the study. Second, we excluded patients intubated with adjunctive devices (e.g., bougie). The use of a bougie may have resulted in a higher rate of successful initial intubation regardless of VL or DL [29,30]. Indeed, our sensitivity analysis demonstrated a consistent inference. Third, with the increased use of VL over the years in Japan [9], there were some differences in patient characteristics between DL and VL groups. While we adjusted patent characteristics and ED visit years, there were potential unmeasured confounding factors (such as the actual skill and experience of each intubator). Fourth, the associations between VL use and higher first-pass success among transitional residents and emergency medicine residents were not significant probably due to the limited sample size in the subgroup analysis. Fifth, although time to intubation may be associated with adverse event rates, it is unclear because we have not collected time to intubation. Lastly, our study sample consisted of academic EDs in Japan. While it is tempting to dismiss the generalizability of our inferences, the observed relationship is clinically plausible [12-15].

## Conclusions

In this multicentre prospective study of 5,261 patients who underwent intubation by resident physicians in EDs, the use of VL was associated with a higher first-attempt success rate compared to that of DL. The use of VL was also associated with fewer overall and minor adverse events. Given the importance of first-pass success in ED patients, our study lends additional support to the use of VL by residents. Lastly, our data should advance further research into the development of optimal airway management strategies, which will, in turn, lead to better outcomes for critically ill patients in EDs.
